# Editorial: Insights of important mammalian viruses: Infection, pathogenesis and drugs

**DOI:** 10.3389/fmicb.2023.1147109

**Published:** 2023-02-21

**Authors:** Yihan Li, Douglas Paul Gladue, Sha Li, Lin Deng, Chao Shen, Patricia V. Aguilar, Ting Wang, Lingbao Kong

**Affiliations:** ^1^Institute of Pathogenic Microorganism and College of Bioscience and Engineering, Jiangxi Agricultural University, Nanchang, China; ^2^Plum Island Animal Disease Center, Agricultural Research Service, United States Department of Agriculture Greenport, Greenport, NY, United States; ^3^Division of Infectious Disease Control, Center for Infectious Diseases, Kobe University Graduate School of Medicine, Kobe, Japan; ^4^College of Life Sciences, Wuhan University, Wuhan, China; ^5^Department of Pathology, University of Texas Medical Branch, Galveston, TX, United States

**Keywords:** mammalian viruses, pathogenesis, virus-host interaction, animal models, epidemiological surveillance

COVID-19, has brought new attention to the importance of new or emerging pathogenic viruses as a major threat to global health. Viruses have evolved multiple mechanisms to counteract host immunity that can be similar or drastically different for various viruses, making it difficult to control viruses using a single countermeasure. To further increase the complexity of controlling viruses, during replication viruses can vary genetically providing the viruses with the ability to overcome a countermeasure. As has been demonstrated by SARS-CoV-2, the viruses can evolve changing from the original target strains. Understanding the molecular mechanisms involved in how different viruses evade the immune system, or how different mutations could cause species shift is of great importance, especially for mammalian viruses. Evasion of immune system and/or genetic evolution can influence the virulence of the viral species, with the potential to cause pandemics. This collection of articles and reviews updates information on the research into the pathogenesis, diagnostic methods and vaccine development for a number of important mammalian viruses, which could be exploited to control the spread of existing viral diseases and prevent future potential virus epidemics.

Viruses are obligate intracellular pathogens and rely on host biological machinery for their own survival. Though different viruses have distinct infection characteristics and pathogenesis characteristics, a deeper understanding of how the virus interacts with the host and in particular virus-host protein-protein interactions can facilitate the control of viral infections. Ruan et al. observed that Enterovirus 71 (EV71) infection caused a significant increase of cellular exosome secretion. They further demonstrated the interaction between EV71 3A and vacuolar protein sorting 25 (VPS25) facilitates exosome biogenesis through the endosomal sorting complex required for transport (ESCRT) pathway, which favors virus replication. Xie et al. studied the underlying mechanism of pseudorabies virus (PRV) infection. They highlighted that porcine DDX56 promotes IFN-β expression through cGAS-STING signaling pathway to inhibit PRV proliferation. Viral infection in pregnancy and vertical transmission that can result in fetal abortions and malformations is an important focus of animal viral research. Trinh et al. found low glucose-induced endoplasmic reticulum (ER) stress may play a key role in increasing rubella virus (RuV) infection and the risk of congenital rubella syndrome (CRS) in early pregnancy.

Animal models have made outstanding contributions to the elucidation of the pathogenesis and transmission mechanisms of human viral diseases. To mimic the symptoms in humans, different animal models have been applied in COVID-19 research, of which the major strengths and weaknesses were comparatively evaluated by Zhao et al. They noted that animal models have shown a great utility in viral studies, yet a credible and reasonable animal model that encompasses all aspects of human COVID-19 has not been found. Depression is a common mental disorder that is gaining increasing interest, but its pathogenesis is poorly understood. Analyzing the core symptoms of depression manifested by specific animal models is an important approach to reveal the underlying mechanisms of depression. Based on viral metagenomics and metabolomics analyses, Duan et al. established a chronic restraint stress (CRS)-induced mouse model of depression to study the relationship between gut virome and depression. Gut virome dyregulation was observed in the CRS mouse model and further investigation revealed that the differential gut virome was strongly associated with neurotransmitter metabolites. Meanwhile this study also pointed out the existing deficiencies of current research, such as lack of clinical data, small sample size and no examination of gut bacteria.

Investigating the occurrence and prevalence of infectious diseases is necessary to control the spread of epidemics and prevent the outbreak of newly emerging viruses. Therefore, the development of rapid, accurate and cost-effective methods to identify viruses is important for epidemiological investigations. In this collection, optimized epidemiologic methods were proposed. Wu et al. accurately classified human adenoviruses (HadVs) by polymerase chain reaction with specially designed primers that target the variable regions of the three major capsid genes span the genome. Hu et al. conducted a detailed seroepidemiological survey of porcine circovirus 4 (PCV4) in Jiangxi Province of China based on an indirect immunosorbent assay (ELISA) with purified His-Cap or His-Rep proteins as the coating antigens.

However, there are certain limitations for standard epidemiological surveillance in predicting the behavior of infectious diseases, as modern transportation has been recognized as the predominant driver of global spread of viruses. To clarify the origin and dispersal routes of bovine leukemia virus (BLV), Nishikaku et al. used phylogenetic and Bayesian phylogeographic analyses. In their study, it was demonstrated that the original BLV came from Asia and zebu cattle were the source of introducing BLV into the taurine cattle. Interestingly, several Asian indigenous bat species shares endogenous delta-retrovirus sequences closely related to BLV lineage II, which further supports the origin of BLV virus in Asia. Ruiz-Saenz et al. raised a warning that African swine fever (ASF) is posing a potential threat to the swine industry in the Americas. With the difficulty of diagnosing ASF and the absence of effective vaccines, there is an urgent need to improve precautionary methods and establish international cooperation for the prevention of ASF spread globally.

In a conclusion, the collection of articles highlights some important advances in virology, that will aid in the understanding of mammalian viruses and potentially be useful in prevention of the next virus pandemic.

## Author contributions

YL: writing—original draft. DG, SL, LD, CS, PA, and TW: writing—review and editing. LK: supervision. All authors contributed to the article and approved the submitted version.

